# Interaction Between *CTLA-4*, *FOXO-3*, and *PTPN-22* Variants and Environmental Factors in Type 1 Diabetes—Observational Association Study

**DOI:** 10.3390/nu17243886

**Published:** 2025-12-12

**Authors:** Edyta Cichocka, Anna Maj-Podsiadło, Sylwia Barbara Górczyńska-Kosiorz, Nikola Szweda-Gandor, Janusz Gumprecht

**Affiliations:** Department of Internal Medicine, Diabetology and Nephrology, Faculty of Medical Sciences in Zabrze, Medical University of Silesia, 40-055 Katowice, Poland; annamaj@sum.edu.pl (A.M.-P.); skosiorz@sum.edu.pl (S.B.G.-K.); nszweda@sum.edu.pl (N.S.-G.); jgumprecht@sum.edu.pl (J.G.)

**Keywords:** environmental factors, genetic factors, metabolic adaptation, type 1 diabetes, MASLD, AITD, diet, Vitamin D, probiotic

## Abstract

**Background**: Immune-regulatory genes such as *CTLA-4*, *FOXO-3*, and *PTPN-22* influence immune tolerance and metabolic adaptation, but their interaction with environmental factors in type 1 diabetes (T1DM) remains unclear. **Methods**: In this observational associated study, we analyzed *CTLA-4* (rs3087243, rs231775), *FOXO-3* (rs2802292, rs9400239), and *PTPN-22* (rs12730735) polymorphisms in 277 adults with T1DM, assessing associations with probiotic and vitamin D use, self-reported dietary patterns, metabolic control, autoimmune thyroid disease (AITD), and metabolic dysfunction-associated steatotic liver disease (MASLD). **Results**: Across the cohort, *CTLA-4* rs3087243 G and *FOXO-3* rs2802292 T alleles were associated with higher AITD risk (*p* = 0.016–0.03), significant in both dominant and additive models. The effect persisted by sex: *CTLA-4* in women and *FOXO-3* in men. Stratified analyses revealed metabolic advantages for *CTLA-4* G and *FOXO-3* T carriers (vegetarian diet, lower HbA1c, stress adaptation). *FOXO-3* rs9400239 T was linked to MASLD (*p* ≈ 0.037–0.041), with similar trends for *CTLA-4* rs231775, stronger in men. Vitamin D supplementation showed protective trends, particularly in *FOXO-3* rs2802292 GG and *CTLA-4* GG/AG carriers. Conversely, probiotic use was associated with higher AITD in *FOXO-3* rs2802292 GT and *CTLA-4* rs3087243 GG genotypes. **Conclusions**: *CTLA-4*, *FOXO-3*, and *PTPN-22* variants may modulate the metabolic and autoimmune response to environmental factors including nutrients in T1DM.

## 1. Introduction

**Figure 1:**
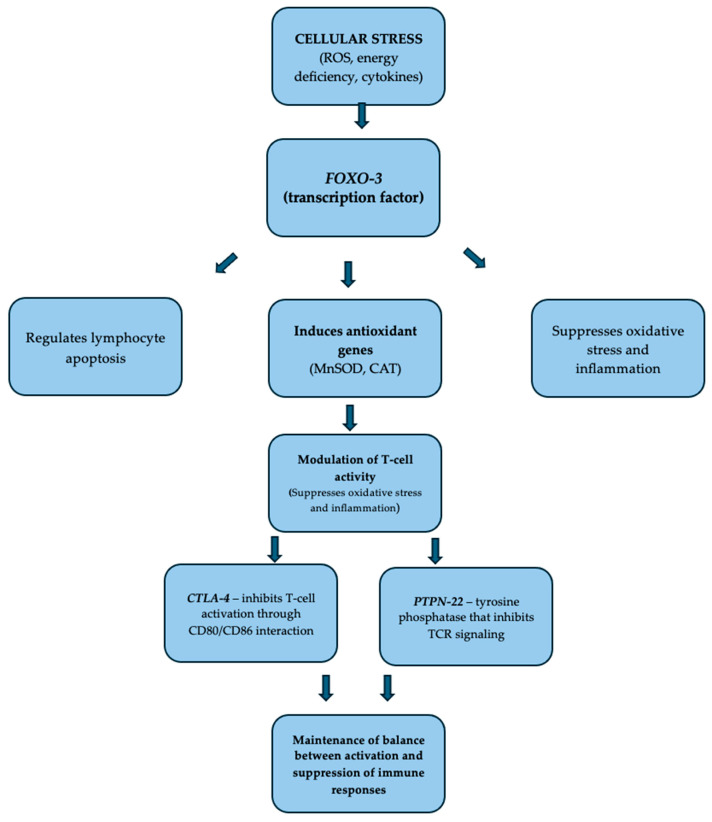
Interplay between *FOXO-3*, *CTLA-4*, and *PTPN-22* in maintaining immune homeostasis (adaptation from [10,11,12]).

Type 1 diabetes (T1DM) is a chronic autoimmune disease characterized by immune-mediated destruction of pancreatic β-cells, leading to absolute insulin deficiency and long-term metabolic imbalance [1,2]. While the genetic background—particularly the HLA complex—remains the strongest determinant of T1DM risk, accumulating evidence indicates that non-HLA immune-regulatory genes and environmental exposures jointly influence disease onset, progression, and clinical phenotype [3,4,5]. In type 1 diabetes, HLA class II alleles—particularly the HLA-DR3-DQ2 and HLA-DR4-DQ8 haplotypes—represent the strongest genetic determinants of disease risk by creating a high-risk HLA background that favors presentation of β-cell antigens. Their peptide-binding grooves stabilize the presentation of key islet autoantigens (e.g., insulin B:9–23, GAD65, IA-2, ZnT8), leading to activation of autoreactive CD4^+^ T cells. At the same time, these alleles present self-antigens inefficiently during thymic selection, allowing diabetogenic T-cell clones to escape deletion. The synergistic effect of the DR3/DR4 genotype further enhances susceptibility, promoting breakdown of tolerance and progression of autoimmune β-cell destruction [6,7]. In turn, among these non-HLA loci, cytotoxic T-lymphocyte-associated protein 4 (*CTLA-4*), forkhead box O3 (*FOXO-3*), and protein tyrosine phosphatase non-receptor type 22 (*PTPN-22*) have emerged as important regulators of immune tolerance and metabolic homeostasis—see Table 1 and Figure 1 [2,8,9,10,11,12]. Table 2 summarizes the characteristics of the analyzed genetic variants together with their clinical interpretation. For this purpose, we used the ClinVar database (NCBI), which is considered one of the most reliable and transparent sources of information on the clinical relevance of genetic variants. ClinVar allows verification of whether a variant has been previously reported in individuals with a given disease phenotype and whether its classification is supported by consistent evidence. This database was chosen because it is widely used in molecular diagnostics and research and serves as a key reference for the classification and interpretation of genetic variants. Variants in selected genes affect immune checkpoint function, T-cell activation, apoptosis, and cytokine signaling, thereby contributing to the balance between tolerance and autoimmunity [8,13], as well as potentially influencing metabolic adaptation. However, the functional consequences of common polymorphisms in these genes are not uniform and may be strongly modulated by environmental and lifestyle factors. In parallel, environmental and nutritional factors—including dietary composition, nutrient intake (e.g., vitamins), and gut microbiota-derived metabolites—are increasingly recognized as modulators of immune and metabolic pathways [13,14]. Nutrients such as fatty acids, vitamins D, antioxidants, and polyphenols may affect immune regulation, oxidative stress responses, and metabolic signaling. Therefore, interaction between genetic predisposition and environmental factors could shape disease phenotype in T1D, influencing not only disease onset but also comorbidities, metabolic control, and adaptation [13,15,16,17]. It is worth mentioning that vitamin D plays an immunomodulatory role partly by downregulating TLR-4 (Toll-like receptor 4) expression through binding of the vitamin D receptor (VDR) to regulatory elements in the TLR4 promoter, thereby reducing pro-inflammatory signaling in innate immune cells [18]. Short-chain fatty acids (SCFAs), particularly butyrate and propionate, also enhance peripheral immune tolerance by promoting FoxP3^+^ regulatory T-cell (Treg) differentiation via histone deacetylase (HDAC) inhibition and GPR43/GPR109A (G protein-coupled receptors) receptor signaling [19,20]. Together, these pathways provide a mechanistic framework linking environmental exposures, gut microbial metabolites, and modulation of inflammatory responses.

Despite these insights, relatively few studies have examined gene and environment interactions in T1DM by combining genetic variants in immune-regulatory genes with detailed environmental/nutritional data [21,22,23,24]. This gap is relevant because genetic variants may predispose to altered immune or metabolic responses, but the manifestation of those predispositions could depend heavily on modifiable factors such as diet, supplementation, or gut microbiota. Understanding these mechanisms may support the development of personalized nutritional strategies aimed at improving metabolic outcomes and modulating autoimmune activity. We selected these mentioned SNPs because they lie in functionally relevant immune-regulatory or metabolic genes (*CTLA-4*, *PTPN-22*, *FOXO-3*), and prior studies have shown associations between these SNPs (or closely linked variants) and autoimmune diseases or metabolic traits (e.g., *FOXO-3* rs2802292 has been linked to metabolic health.

The aim of this study was to investigate whether common variants in *CTLA-4* (rs3087243, rs231775), *FOXO-3* (rs2802292, rs9400239), and *PTPN-22* (rs12730735) modify the association between environmental/nutritional factors (dietary patterns, vitamin D supplementation, probiotic use) and metabolic or autoimmune-related outcomes (metabolic control, autoimmune thyroid disease (AITD), metabolic dysfunction-associated steatotic liver disease (MASDL) in adults with T1D. By focusing on these interactions, we aimed to clarify how genetic predisposition and modifiable lifestyle factors jointly influence immune tolerance and metabolic adaptation in T1D.

**Table 2:** Genetic characteristics of the analyzed genetic variants.

SNP ID (Gene)	Reference/ Alternative Allele	Genomic Location (hg38)	HGVS Nomenclature	Minor Allele Frequency (EUR)	ClinVar Accession	Associated Phenotypic or Disease Trait
rs3087243 (*CTLA-4*)	A/G	chr2:203874196	NG_011502.1:g. 11411G > A	0.35	RCV001515646.7	T1D, AID [1,2]
rs231775 (*CTLA-4*)	G/A	chr2:203867991	NG_011502.1:g. 5206A > G	0.45	RCV001255201.1	T1D, general autoimmunity [1,2,8]
rs12730735 (*PTPN-22*)	T/C	chr1:113838835	NG_007403.2:g. 331 + 713T > C	0.25	Not listed in ClinVar	AITD, region-specific effects [2,3]
rs2802292 (*FOXO-3a*)	T/G	chr6:108587315	NG_158842.1:g. 239G > T	0.40	Not listed in ClinVar	Longevity, immune system regulation [22]
rs9400239 *(FOXO-3a*)	C/T	chr6:108656460	NG_012124.2:g. 62345C > T	0.30	Not listed in ClinVar	Longevity, metabolic characteristics [22]

Abbreviations: ClinVar—public archive with free access to reports on the relationships between human genetic variations and phenotypes; HGVS—Human Genetic Variation Society; hg—human genome; SNP—single-nucleotide polymorphism; rs—reference SNP cluster ID; A—adenine; C—cytosine; T—thymine; G—guanine; chr—chromosome; *CTLA-4*—cytotoxic T-lymphocyte-associated protein 4; *FOXO-3*—forkhead box O3; *PTPN-22*—protein tyrosine phosphatase non-receptor type 22; T1D—type 1 diabetes; AID—autoimmune disease.

## 2. Materials and Methods

### 2.1. Study Group

The study protocol was approved by the Bioethics Committee of the Medical University of Silesia, Poland (PCN/0022/KB1/104/19). All eligible participants provided written informed consent prior to enrolment in the study. The study cohort consisted of 277 consecutive adults with T1D who were admitted to the Department of Internal Medicine, Diabetology and Nephrology or were under the care of the Diabetes Outpatient Clinic, Medical University of Silesia between 2021 and 2024 (Zabrze, Poland). Demographic and anthropometric data, including sex, age, body weight, and height, were collected. Clinical and environmental information was also recorded, including diabetes duration and therapy, all comorbidities, particularly AITD and MASLD, and medication use.

MASLD (Metabolic Dysfunction-Associated Steatotic Liver Disease), according to the applicable guidelines, was defined as liver fat accumulation (steatosis) in someone with at least one cardiometabolic risk factor, such as obesity, type 2 diabetes, hypertension, or high triglycerides, while excluding significant alcohol intake [25].

Participants provided additional data on lifestyle and nutritional factors, including smoking habits, alcohol consumption, physical activity, probiotic and vitamin D supplementation (defined as supplementation at least 2000 IU per day), and self-reported dietary patterns (defined as following a balanced diet with restricted intake of simple sugars and, in some individuals, also lactose or gluten-free diets, or vegetarianism). Metabolic control parameters were also assessed.

Perceived stress was evaluated using the validated Perceived Stress Scale–10 (PSS-10). Participants completed the 10-item questionnaire, and total scores were calculated according to the standardized scoring protocol. In accordance with established cutoffs, a score >27 was defined as high perceived stress, and this categorical variable was used in subsequent statistical analyses.

Exclusion criteria included the presence of other types of diabetes or refusal to provide informed consent. All procedures were conducted in accordance with the ethical standards of the institutional research committee and the Declaration of Helsinki.

### 2.2. Biochemical and Genetic Analyses

Venous blood samples (2 × 4.9 mL) were collected from each participant. Glycated hemoglobin (HbA1c) was measured using high-performance liquid chromatography (HPLC). Thyroid function and autoimmunity were assessed via thyroid-stimulating hormone (TSH), thyroid peroxidase (TPO) antibodies, and antithyroglobulin (TG) antibodies, with positivity defined as TPO > 30 IU/mL and TG > 4.11 IU/mL.

SNPs associated with autoimmune conditions were identified from the literature and the NCBI SNP database. Five SNPs were selected for genotyping: *CTLA-4* (rs3087243, rs231775), *PTPN-22* (rs12730735), and *FOXO-3a* (rs2802292, rs9400239). We selected these mentioned SNPs because they lie in functionally relevant immune-regulatory or metabolic genes (*CTLA-4*, *PTPN-22*, *FOXO-3*), and prior studies have shown associations between these SNPs (or closely linked variants) and autoimmune diseases or metabolic traits (e.g., *FOXO-3* rs2802292) has been linked to metabolic health. Genomic DNA was extracted from erythrocytes using the MagCore Genomic DNA Whole Blood Kit (RBC Bioscience Corp., New Taipei City, Taiwan) and stored at −20 °C. Genotyping was performed using TaqMan probes on a Roche LifeCycler 96 qPCR system (Roche, Basel, Switzerland), with two blank controls included per plate. To ensure the reliability of the SNP analyses, we repeated genotyping for a randomly selected 10–15% of DNA samples. The repeatability of the results obtained was 100%, which confirmed consistency with previously obtained genotyping analyses. Allelic frequencies were calculated for all five loci.

### 2.3. Statistical Analysis

Statistical analyses were performed in R (version 2024.04.2+764.pro1) via RStudio (version 2024.04.1+748). Categorical variables were expressed as counts and percentages, and continuous variables as mean ± SD or median (Q1–Q3), depending on distribution. The normality of the distribution was evaluated based on histograms and Q–Q plots. Associations between categorical variables were evaluated using the chi-square or Fisher’s exact test, as appropriate. Continuous variables were compared between two independent groups using Student’s *t*-test for normally distributed data or the Wilcoxon rank-sum (Mann–Whitney) test for non-parametric data. For continuous outcomes across multiple genotype groups, differences were assessed using one-way ANOVA for normally distributed data or the Kruskal–Wallis test otherwise, followed by Bonferroni-corrected post hoc comparisons when applicable.

Allele distributions were checked for Hardy–Weinberg equilibrium and compared with Central European population frequencies (The proportion test). In the additive genetic model, each additional copy of the minor allele was assumed to have a linear (dose-dependent) effect on the outcome variable. Genotypes were coded as 0, 1, and 2, corresponding to the number of minor alleles. The regression coefficient therefore represented the change in log-odds (for logistic regression) or in mean value (for linear models) per one additional minor allele.

In the dominant model, carriers of at least one minor allele were grouped together and compared with homozygotes for the major allele (e.g., AA vs. AG + GG). This model tested whether the presence of the minor allele, regardless of dosage, influenced the outcome.

Univariate logistic regression was used to estimate odds ratios (ORs) and 95% confidence intervals (CIs) for binary traits. Multivariable logistic regression models were constructed to examine SNP interactions with sex, disease duration, and metabolic parameters, including effects on glycaemic control. Model fit was assessed using the Hosmer–Lemeshow goodness-of-fit test.

Interactions between genetic variants and environmental or lifestyle factors (e.g., stress exposure, probiotic use, physical activity, or dietary habits) were explored using two-way ANOVA or the Scheirer–Ray–Hare test for non-parametric data. The Benjamini–Hochberg false discovery rate (FDR) correction was applied to control for multiple testing and to reduce the probability of type I errors arising from the large number of SNP comparisons. All tests were two-tailed, and statistical significance was set at *p* < 0.05.

## 3. Results

The clinical characteristics of the study population are summarized in Table 2. The cohort included 277 adults with T1D, of whom 65.7% were female. The mean age was 33.13 ± 12.47 years, with a median diabetes duration of 12 (4; 19) years. Most participants were treated with multiple daily insulin (MDI) injections (59.5%), and 58.8% used continuous glucose monitoring (CGM) systems. The average HbA1c level was 8.15 ± 2.04%, with no significant differences between sexes. Additionally, the prevalence of chronic diabetes complications and other comorbidities, including AITD and MASLD, and data on individual environmental factors was assessed (Table 3).

The frequencies of alleles and genotypes for the analyzed polymorphisms are summarized in Supplementary Table S1. Genotype distributions complied with the Hardy–Weinberg equilibrium (*p* > 0.01). A statistically significant variation was observed in the allele frequencies of the rs3087243 polymorphism within the *CTLA-4* gene when compared with data from the Central European population. In contrast, the remaining analyzed polymorphisms showed no significant differences relative to the Central European reference group (Supplementary Table S2).

We evaluated the association between selected SNPs and the risk of AITD. In the overall population, *CTLA-4* rs3087243 and *FOXO-3* rs2802292 were significantly associated with increased AITD risk under the dominant model (carriers of ≥1 risk allele vs. non-carriers; *p* = 0.016 and *p* = 0.017, respectively). Additionally, *FOXO-3* rs2802292 showed a significant association under the additive model (0–1–2 risk alleles; *p* = 0.030). When stratified by sex, male carriers of the *FOXO-3* rs2802292 risk allele also had an increased risk of AITD (*p* = 0.043) (Table 4 and Table 5). After applying the Benjamini–Hochberg false discovery rate (FDR) correction for multiple testing, the strength of evidence for the initially observed associations between SNPs and AITD was attenuated. The adjusted *p*-values clustered around the 0.04–0.05 range (*CTLA-4* rs3087243 and *FOXO-3* rs2802292 in the dominant model), indicating borderline rather than robust statistical significance. Importantly, the effects did not disappear entirely after correction; however, their magnitude and statistical support were weakened. Therefore, the detected signals should be interpreted as exploratory and requiring independent replication.

We next examined the relationship between SNPs and MASLD. In males, carriers of at least one risk allele of *FOXO-3* rs9400239 had an increased risk of MASLD under the dominant model (*p* = 0.037). Additionally, *PTPN-22* rs12730735 C allele carriers had a lower mean MASLD risk under the additive model, suggesting a potential protective effect (mean difference = −0.20; *p* = 0.049) (Table 6).

The distribution of selected SNP genotypes was assessed in participants with MASLD and vitamin D supplementation. For *CTLA-4* rs3087243, the AA genotype was present in 32.0% of individuals, GG in 59.5%, and AG in 55.1% among those with MASLD and vitamin D supplementation (pχ^2^ = 0.042). Similarly, *FOXO-3* rs2802292 genotypes showed significant associations, with GG at 39.1%, GT at 56.8%, and TT at 61.3% in participants with MASLD and vitamin D supplementation (pχ^2^ = 0.047) (Table 7).

The convergence of *p*-values obtained from the χ^2^ test and Fisher’s exact test in both the dominant and recessive models reflects the stability of the observed associations, suggesting that the effect is primarily driven by the difference between carriers and non-carriers of the minor allele (Table 8).

We also evaluated the relationship between SNPs and probiotic use. The *FOXO-3* rs2802292 T allele was significantly associated with higher probiotic use under the additive model (mean difference = 0.33; *p* = 0.037), which may have potential implications for clinical relevance (Table 9).

We investigated interactions between genetic variants and stress exposure on probiotic use. In the overall cohort, carriers of at least one risk allele of *CTLA-4* rs3087243 and *CTLA-4* rs231775 were more likely to use probiotics under stress (OR = 1.80, 95% CI: 1.06–3.05; *p* = 0.034 and OR = 2.08, 95% CI: 1.15–3.76; *p* = 0.018, respectively). For *FOXO-3*, carriers of the rs2802292 risk allele also had increased probiotic use under stress (OR = 1.86, 95% CI: 1.07–3.22; *p* = 0.029), while non-carriers of *FOXO-3* rs9400239 showed higher probiotic use under stress in both the total cohort (OR = 2.40, 95% CI: 1.08–5.33; *p* = 0.048) and in females (OR = 3.06, 95% CI: 1.17–8.00; *p* = 0.034) (Table 10).

For a comprehensive analysis, we also assessed the relationships between the studied genetic variants and adherence to vegetarian diets, as well as alcohol consumption. All results obtained showed no statistical significance (*p* > 0.05), which does not allow us to confirm the existence of an association between the analyzed polymorphisms and these environmental factors in the studied population. In our analysis using standard genetic association formulas, we show that the study had ~80% power to detect moderate effects (OR ≥ 1.8) for SNPs with MAF ≥ 0.25, with lower power for subgroup analyses.

## 4. Discussion

In this study, we examined the interplay of selected genetic polymorphisms (in *CTLA-4*, *FOXO-3*, and *PTPN-22*) with AITD, MASLD, probiotics, and vitamin D supplementation and stress exposure in a cohort of patients with T1D. Consistent with previous studies, thyroid disorders were the most prevalent autoimmune conditions in our T1D cohort, affecting over 30% of participants [26,27]. Our results also indicate that *CTLA-4* rs3087243 and *FOXO-3* rs2802292 are significantly associated with increased AITD risk under both dominant and additive genetic models. These findings align with prior evidence highlighting the role of *CTLA-4* in immune regulation and *FOXO-3* in inflammatory responses and immune aging.

Sex-stratified analysis revealed that male carriers of *FOXO-3* rs2802292 also have an elevated risk of AITD, suggesting possible sex-specific genetic effects, which warrant further investigation. This finding is consistent with meta-analytic evidence that *CTLA-4* CT60 (rs3087243) confers susceptibility to AITD across multiple ethnic groups (OR ~1.26 for HT; OR ~1.45 in Asians) as shown by Ni et al. in 2014 [28]. Although *FOXO-3* is more widely studied in the context of longevity and metabolic regulation rather than thyroid autoimmunity, its role in stress response and immune modulation (e.g., via chromatin hubs and enhancer regulation) has been described [29].

Our findings also suggest that the *PTPN-22* rs12730735 polymorphism may play a protective role in the development of MASLD in our cohort, and male carriers of *FOXO-3* rs9400239 showed increased susceptibility, emphasizing a potential link between *PTPN-22* and *FOXO-3* variants and metabolic liver dysfunction in T1D.

The emerging literature supports the role of *FOXO-3* variants in metabolic regulation and liver fat accumulation; for example, in a Southern Italian cohort the *FOXO-3* rs2802292 G allele was found to influence food intake and MASLD risk [30,31]. Taken together, our results point to a dual involvement of immune regulation and metabolic pathways via *CTLA-4*, *PTPN-22*, and *FOXO-3* variants in this T1D sample.

In addition to disease associations, our study observed significant genotype distribution differences in relation to vitamin D supplementation and MASLD occurrence. *CTLA-4* rs3087243 genotype distributions (AA, GG, AG) were associated with differing proportions of MASLD or vitamin D use (pχ^2^ = 0.042). The interplay between genotype and nutrient behavior supports the concept of the nutrigenetic idea that genetic background modulates responses to nutrients or supplementation [32]. Although studies specifically linking *CTLA-4* or *FOXO-3* to vitamin D or probiotic use are limited, our observation of higher probiotic use in carriers of *FOXO-3* rs2802292 (mean difference = 0.33; *p* = 0.037) may suggest a potential genotype-related influence on health-related behavior. However, this finding should be interpreted with caution. An important extension of our findings concerns gene–environment interactions. We found that under stress exposure, carriers of *CTLA-4* rs3087243 and rs231775, and carriers or non-carriers of *FOXO-3* variants, had increased probiotic use. For example, *CTLA-4* rs231775 carriers had OR = 2.08 (95% CI 1.15–3.76; *p* = 0.018).

These results reinforce the possibility that stress exposure, probiotic behavior, and genotype may operate in concert; stress can influence gut-microbiome composition and immune regulation, probiotics also influence the microbiome and immune function, and genetic variants such as those in *CTLA-4* and *FOXO-3* can modulate these pathways. This gene-nutrient-environment connection is increasingly recognized as a promising paradigm in precision nutrition research [33,34,35]. Our findings could have important implications for precision nutrition and personalized medicine. However, these results should be interpreted with caution due to the analyses of many comparisons. Identifying T1D patients carrying at-risk alleles in *CTLA-4* or *FOXO-3* may enable targeted interventions such as optimized vitamin D supplementation, tailored probiotic regimens, stress management strategies, and lifestyle modifications to reduce AITD or MASLD risk. Importantly, genotype alone does not determine outcome—nutrient behavior, supplementation, and environmental exposures shape phenotype expression.

This study has several limitations. First, its cross-sectional design precludes causal inference regarding the observed associations between genetic variants, environmental factors, and clinical outcomes. Second, although the overall sample size was sufficient to detect moderate effects in the total cohort, it was relatively limited for stratified analyses (e.g., sex-specific models—with particularly small numbers of males—or gene–environment interaction strata), which reduces statistical power and increases the risk of type II error. Third, environmental and nutritional exposures were assessed using self-reported measures and were restricted mainly to stress and supplementation behaviors; other relevant factors that may modulate genotype–phenotype associations were not captured. Moreover, we did not collect detailed quantitative dietary data, objective nutritional biomarkers (e.g., metabolomic profiles or specific nutrient biomarkers), or microbiome profiles, and vitamin D status was inferred from self-reported supplementation rather than serum 25(OH)D concentrations (we plan to include laboratory measurements of vitamin D levels in future analyses). Finally, although the single-center design ensured highly standardized clinical assessment and laboratory procedures, it may limit the generalizability of the results. Larger, multicenter, longitudinal studies with comprehensive dietary quantification, biomarker measurements, and microbiome analyses are needed to validate and extend our observations. A key unmet need is the quantitative characterization of diet and nutrient–gene interactions in T1D. In our study, dietary information was limited to self-reported patterns (e.g., vegetarian vs. non-vegetarian), without detailed quantification of macronutrient or micronutrient intake, meal timing or dietary quality indices. Future work should incorporate standardized dietary assessment tools (such as weighed food records or validated food frequency questionnaires), combined with metabolomic profiling and microbiome analyses, to capture nutrient-derived metabolites and gut microbial products. Integrating these layers with genetic data in formal nutrient–gene interaction models (e.g., genotype-stratified regression, multi-omics integration) will be crucial to elucidate how specific nutrients, microbial metabolites and genetic variants jointly shape immune and metabolic phenotypes in T1D.

Therefore, while our findings suggest intriguing associations and generate hypotheses for personalized nutritional/therapeutic strategies, they should be regarded as exploratory.

## 5. Conclusions

Our study provides preliminary evidence that common variants in *CTLA-4*, *PTPN-22*, and *FOXO-3* may contribute to the heterogeneity of autoimmune and metabolic comorbidities in T1D. The associations identified appear to depend, at least in part, on environmental and lifestyle exposures, indicating that genetic susceptibility interacts with modifiable factors rather than acting in isolation. These findings highlight the potential relevance of gene–nutrient and gene–environment interactions in shaping clinical expression of T1D.

Given the exploratory and observational character of the study, limited statistical power in subgroup analyses, and reliance on self-reported environmental data, the results should be interpreted cautiously. Nonetheless, they generate hypotheses suggesting that nutrition- and microbiota-oriented interventions, when aligned with genetic background, may hold potential for improving the management of T1D-related comorbidities.

## 6. Future Perspectives

Future research should validate these findings in larger, multi-center cohorts and, ideally, through longitudinal designs capable of capturing temporal relationships among genetic predisposition, environmental exposures, and clinical outcomes. To advance understanding of gene–nutrient interactions, upcoming studies should integrate detailed dietary quantification, standardized assessment of supplementation and stress, biomarker-based nutritional profiling, and microbiome analyses.

In the long term, genotype-stratified interventional trials will be essential to determine whether personalized nutritional, microbial, or lifestyle-based strategies can effectively reduce the risk or severity of autoimmune and metabolic complications in T1D. Such work is necessary before translating preliminary associations into clinical or public-health recommendations.

## Figures and Tables

**Table 1:** Functional roles and interactions of *CTLA-4*, *PTPN-22*, and *FOXO-3* in immune tolerance and metabolic balance.

Gene	Main Function	Relationship/Interaction with Other Genes/Pathways
*CTLA-4*	Negative regulator of T-cell activation; limits immune responses by binding CD80/CD86 (B7) on antigen-presenting cells (APCs), thereby inhibiting T-cell costimulation and downstream TCR signaling [2,8,9,10]	Serves as an immune checkpoint that restrains T-cell activation—functionally complementary to intracellular regulators such as *PTPN-22* [2,8,9,10].
*PTPN-22*	A tyrosine phosphatase that downregulates T-cell receptor (TCR) signaling, thereby attenuating activation and effector responses of T cells (and other immune cells). Genetic variants (e.g., R620W) are associated with increased risk of autoimmune diseases [2,8,9,11]	Acts independently of *CTLA-4* but in a complementary regulatory way to limit T-cell activation and immune responses [2,8,9,11].
*FOXO-3*	A transcription factor involved in oxidative stress response (antioxidant gene expression), apoptosis, and metabolic and immune regulation [2,8,9,12].	*FOXO-3* (specifically *FOXO-3a*) activation in dendritic cells (DCs) can induce antioxidant defenses (e.g., superoxide dismutase), reduce oxidative stress, and support tolerogenic immune responses. This may modulate the function of immune-regulatory pathways, possibly indirectly affecting *CTLA-4*—dependent tolerance [2,8,9,12].

Abbreviations: APC—antigen-presenting cell; TCR—T-cell receptor; *CTLA-4*—cytotoxic T lymphocyte-associated protein 4; *PTPN-22*—protein tyrosine phosphatase non-receptor type 22; *FOXO-3*—forkhead box O3; DC—dendritic cell.

**Table 3:** General characteristics of the study population.

Variable	N (%)	Summary Statistic *
Sex	277	Women: 182 (65.7%), Men: 95 (34.3%)
Age, years	277	33.13 ± 12.47
BMI, kg/m^2^	277	24.45 ± 4.35
T1D	277	
T1D duration, years	277	12 (4; 19)
T1D treatment	277	MDIs: 165 (59.6%), CSII: 112 (40.4%)
CGM use	163 (58.8%)	
Type of CGM device		FreeStyle Libre 1 or 2: 119 (42.9%), Dexcom 5 or 6: 10 (3.6%), Guardian-3: 34 (12.2%)
HbA1c, %	277	8.15 ± 2.04
Chronic diabetes complications		
Retinopathy	58 (20.9%)	
Nephropathy	16 (5.7%)	
Neuropathy	32 (11.5%)	
Thyroid diseases	91 (32.8%)	
Thyroid disease duration, years	91 (32.8%)	6 (2; 11)
Graves’ disease	4 (4.4%)	
Hypothyroidism	73 (80.2%)	
Hashimoto’s disease	58 (63.3%)	
Nodular goiter	18 (19.8%)	
TSH, µIU/mL	277	1.76 (1.22; 2.38)
TPOAb, IU/mL	277	14.98 (10.77; 39.85)
TGAb, IU/mL	277	1.87 (1.02; 11.98)
Other autoimmune diseases		
Vitiligo	6 (2.1%)	
Celiac disease	9 (3.2%)	
Psoriasis	3 (1.1%)	
Rheumatoid arthritis	3 (1.1%)	
Multiple sclerosis	2 (0.7%)	
Systemic lupus erythematosus	1 (0.4%)	
Addison’s disease	1 (0.4%)	
Other diseases		
Ulcerative colitis	3 (1.1%)	
Atopic dermatitis	31 (11.2%)	
Asthma	12 (4.3%)	
Metabolic dysfunction-associated steatotic liver disease (MASLD)	27 (9.7%)	
Irritable bowel syndrome	7 (2.5%)	
Hypertension	53 (19.1%)	
Ischemic heart disease	7 (2.5%)	
Dyslipidemia	14 (5.0%)	
Polycystic ovary syndrome (PCOS)	19(10.6%)	
Endometriosis	5 (2.8%)	
Family history		
Thyroid diseases	97 (35.0%)	
Diabetes (T1D, T2D)	163 (58.8%)	
Environmental factors		
Smoking and alcohol consumption		
Past or present smoking	221 (79.8%)	
Pack-year history of smoking, years	221	11 (5; 20)
Alcohol consumption (at least once a week)	40 (14.4%)	
Diet		
Gluten-free diet	9 (3.28%)	
Lactose-free diet	3 (1.09%)	
Vegetarian diet	39 (14%)	
Others		
Vitamin D supplementation (at least 2000 IU daily)	106 (38.27%)	
Probiotic supplementation	104 (37.55%)	
High perceived stress (>27 in PSS-10)	151 (54.51%)	

* Continuous variables are presented as mean ± standard deviation for normally distributed data or median (Q1; Q3) for non-normal distribution. Categorical variables are presented as N (%). Abbreviations: T1D—type 1 diabetes; CGM—continuous glucose monitoring; HBA1c—glycated hemoglobin; BMI—body mass index; TPOAb—thyroid peroxidase antibodies; TGAb—thyroglobulin antibodies; TSH—thyroid-stimulating hormone; PSS—perceived stress scale.

**Table 4:** Association between selected SNPs and the risk of AITD.

Gene/SNP	Risk Allele	Genetic Model	n_0_	n_1_	Mean (0)	Mean (1)	Δ (1–0)	*p*	*p* (FDR)
*CTLA-4* rs3087243 (all)	G	dominant (0 vs. ≥1)	25	248	0.32	0.57	0.25	0.016	0.04
*FOXO-3* rs2802292 (all)	T	additive (0/1/2)	123	151	1.02	1.21	0.18	0.030	0.05
		dominant (0 vs. ≥1)	46	228	0.39	0.58	0.19	0.017	0.04
*FOXO-3* rs9400239 (male)	T	dominant (0 vs. ≥1)	30	64	0.03	0.19	0.15	0.045	0.052
*FOXO-3* rs2802292 (male)	T	dominant (0 vs. ≥1)	17	77	0.24	0.51	0.27	0.043	0.051

**Table 5:** Significant associations between SNPs and AITD and MASLD.

Endpoint	Stratum	SNP	Genetic Model	Contrast	Effect	*p*
AITD	ALL	*CTLA-4* rs3087243	dominant	0 vs. ≥1	↑ in group = 1	0.016
AITD	ALL	*FOXO-3* rs2802292	dominant	0 vs. ≥1	↑ in group = 1	0.017
			additive	0–1–2	↑ in group = 1	0.030
AITD	Male	*FOXO-3* rs2802292	dominant	0 vs. ≥1	↑ in group = 1	0.043
MASLD	Male	*FOXO-3* rs9400239	dominant	0 vs. ≥1	↑ in group = 1	0.037

↑ indicates an increase.

**Table 6:** Association between selected SNPs and the risk of MASLD.

Gene/SNP	Risk Allele	Genetic Model	n_0_	n_1_	Mean (0)	Mean (1)	Δ (1–0)	*p*
*PTPN22* rs12730735	C	Additive (0/1/2)	245	27	0.53	0.33	−0.2	0.049

**Table 7:** Genotype distribution of selected SNPs in relation to MASLD and vitamin D supplementation (χ^2^ test).

Gene/SNP	Genotype	Yes (*n*)	Yes (%)	No (*n*)	No (%)	Total (*n*)	*p* (Global)
*CTLA-4* rs3087243	AA/GG/AG	8/72/70	32.0/59.5/55.1	17/49/57	68.0/40.5/44.9	25/121/127	0.042
*FOXO-3* rs2802292	GG/TT/GT	18/49/84	39.1/61.3/56.8	28/31/64	60.9/43.2/38.8	46/80/148	0.047

**Table 8:** Genotype distribution of selected SNPs in relation to MASLD and vitamin D supplementation (Fisher’s test).

Gene/SNP	Genotype Grouping	Yes (*n*, %)	No (*n*, %)	*p*
*CTLA-4* rs3087243	AG + GG vs. AA	142 (57.3%) vs. 8 (32.0%)	106 (42.7%) vs. 17 (68.0%)	0.016
*CTLA-4* rs3087243	GG vs. AA + AG	72 (59.5%) vs. 78 (51.3%)	49 (40.5%) vs. 74 (48.7%)	0.042
*FOXO-3* rs2802292	GT + TT vs. GG	133 (58.3%) vs. 18 (39.1%)	95 (41.7%) vs. 28 (60.9%)	0.017
*FOXO-3* rs2802292	TT vs. GG + GT	49 (61.3%) vs. 102 (52.6%)	31 (38.8%) vs. 92 (47.4%)	0.047

**Table 9:** Association between selected SNPs and the probiotic use.

Gene/SNP	Risk Allele	Genetic Model	n_0_	n_1_	Mean (0)	Mean (1)	Δ (1–0)	*p*
*FOXO-3* rs2802292	T	Additive (0/1/2)	73	21	0.96	1.29	0.33	0.037

**Table 10:** Significant gene–environment interactions.

Exposure	Outcome	Stratum	SNP	Genotype Group (Dominant Model)	a (x = 1, y = 1)	b (x = 1, y = 0)	c (x = 0, y = 1)	d (x = 0, y = 0)	*p* (Fisher)	OR	OR_LCL	OR_UCL
stress	probiotics	all	*CTLA-4* rs3087243	Carrier (≥1 risk)	57	77	33	80	0.034	1.795	1.06	3.05
stress	probiotics	all	*CTLA-4* rs231775	Carrier (≥1 risk)	47	56	27	67	0.018	2.083	1.15	3.76
stress	probiotics	all	*FOXO-3* rs2802292	Carrier (≥1 risk)	56	69	31	71	0.029	1.859	1.07	3.22
stress	probiotics	all	*FOXO-3* rs9400239	Non-carrier (0)	28	28	15	36	0.048	2.4	1.08	5.33
stress	probiotics	female	*FOXO-3* rs9400239	Non-carrier (0)	22	18	10	25	0.034	3.056	1.17	8.0

## Data Availability

The original contributions presented in this study are included in the article/Supplementary Materials. Further inquiries can be directed to the corresponding author.

## Supplementary Materials

The following supporting information can be downloaded at https://www.mdpi.com/article/10.3390/nu17243886/s1, Table S1. The allele and genotype frequencies of the analyzed polymorphisms. Table S2. The allele distribution in the study population compared to that in the Central European population.
