# Supplementary material for: Interaction Between CTLA-4, FOXO-3, and PTPN-22 Variants and Environmental Factors in Type 1 Diabetes—Observational Association Study
Source: Nutrients. 2025 Dec 12;17(24):3886. doi: 10.3390/nu17243886 (PMC12735596; doi:10.3390/nu17243886)
Supplement: Supplementary file 1 [file nutrients-17-03886-s001.zip › nutrients-4026495-supplementary.pdf]

Table S1. The allele and genotype frequencies of the analyzed polymorphisms. HWE—Hardy-Weinberg equilibrium

n = 277		n	%	95% CI		HWE *p*
CTLA-4 rs3087243	AA	25	9.2%	5.8%	12.6%	0.36
	AG	127	46.5%	40.6%	52.4%	
	GG	121	44.3%	38.5%	50.2%	
CTLA-4 rs231775	AA	25	9.2%	5.8%	12.6%	0.54
	AG	127	46.5%	40.6%	52.4%	
	GG	121	44.3%	38.5%	50.2%	
PTPN-22 rs12730735	TT	150	54.9%	49.1%	60.8%	0.68
	CT	107	39.2%	33.4%	44.9%	
	CC	16	5.9%	3.1%	8.6%	
*FOXO-3*A rs2802292	TT	80	29.2%	23.8%	34.5%	0.13
	GT	148	54.0%	48.1%	59.9%	
	GG	46	16.8%	12.4%	21.2%	
*FOXO-3*A rs9400239	CC	104	38.1%	32.4%	43.8%	0.13
	CT	141	51.6%	45.8%	57.5%	
	TT	28	10.3%	6.7%	13.8%	

Table S2. The allele distribution in the study population compared to that in the Central European population. * Ensembl, CE—Central Europe (The proportion test).

	Frequency in the Study Population	95% CI		Frequency in the General Population (CE) *	*p*
CTLA-4 rs3087243					
A	33%	27.5%	38.5%	48%	0.04
G	67%	61.5%	72.5%	52%	
CTLA-4 rs231775					
A	54%	48.1%	59.9%	63%	0.25
G	46%	40.1%	51.9%	37%	
PTPN-22 rs12730735					
C	25%	19.9%	30.1%	29%	0.63
T	75%	69.9%	80.1%	71%	
*FOXO-3*A rs2802292					
G	44%	38.1%	49.8%	35%	0.25
T	56%	50.1%	61.8%	65%	
*FOXO-3*A rs9400239					
T	36%	30.3%	41.6%	27%	0.22
C	64%	58.3%	69.6%	73%	
